# Empathy is positively associated with compassion fatigue among nurses working in neonatal and pediatric intensive care: A cross-sectional study

**DOI:** 10.23938/ASSN.1171

**Published:** 2026-08-19

**Authors:** Birsen Mutlu, Zeynem Yildirim Balkan, Esra Cumur Başkir

**Affiliations:** 1 İstanbul University-Cerrahpasa Florence Nightingale Faculty of Nursing Department of Pediatric Nursing Istanbul Turkey; 2 Namık Kemal University Faculty of Health Sciences Department of Nursing Tekirdağ Turkey; 3 İstanbul University-Cerrahpasa Institute of Graduate Studies Istanbul Turkey

**Keywords:** Empathy, Compassion Fatigue, Nurses, Paediatric, Intensive Care Units, Neonatal, Empatía, Desgaste por Empatía, Enfermeras, Unidades de Cuidados Intensivos Pediátricos, Unidades de Cuidados Intensivos Neonatales

## Abstract

**Background:**

This study examined the association between empathy and compassion fatigue among nurses working in neonatal and pediatric intensive care units.

**Methods:**

A cross-sectional study was conducted among nurses employed in neonatal and pediatric intensive care units at three hospitals in Turkey. Sociodemographic and occupational data were collected, and participants completed the Jefferson Scale of Empathy and the Compassion Fatigue Short Scale. Pearson’s correlation and linear regression analyses were performed to examine the association between empathy, compassion fatigue, and other study variables.

**Results:**

Sixty-five nurses participated in the study, representing 90.3% of the target population. Mean empathy and compassion fatigue scores were 79.77 ± 8.69 and 52.09 ± 20.44, respectively. A moderate positive correlation was observed between empathy and compassion fatigue (r = 0.484). Empathy explained 22.2% of the variance in compassion fatigue (R² = 0.222; B = 1.139; 95% CI: 0.621 - 1.657; *p* < 0.001). Nurses who had voluntarily chosen nursing reported significantly lower compassion fatigue scores than those who had not (49.14 ± 19.39 vs 61.93 ± 21.42; p = 0.032). Working more than 50 hours per week was associated with higher empathy scores (84.06 ± 11.58 vs 78.82 ± 6.46; p = 0.046), but not with compassion fatigue.

**Conclusions:**

Higher empathy associates with greater compassion fatigue among nurses working in neonatal and pediatric intensive care units. Voluntarily choosing nursing associates with lower compassion fatigue, whereas longer working hours associate with higher empathy.

## RESUMEN

**Fundamento:**

El objetivo es analizar la relación entre empatía y fatiga por compasión en personal de enfermería de unidades de cuidados intensivos neonatales y pediátricos, y su relación con otras variables.

**Métodos:**

Estudio transversal realizado en personal de enfermería de cuidados intensivos neonatales y pediátricos de tres hospitales turcos. Se recogieron características sociodemográficas y profesionales, y se administraron la Escala de Empatía de Jefferson y la Escala Breve de Fatiga por Compasión. Se analizó la relación de empatía y fatiga por compasión con las distintas variables, y la relación entre empatía y fatiga por compasión mediante correlación y regresión lineal.

**Resultados:**

Participó el 90,3% del personal (n=65). La edad media fue 31,88 (rango = 23-47) y el 98,5 eran mujeres. La puntuación media de empatía fue 79,77 ± 8,69 y la de fatiga por compasión fue 52,09 ± 20,44. Ambas variables mostraron una correlación positiva, moderada y significativa (r = 0,484). La empatía predijo significativamente la fatiga por compasión (R² = 0,222; B = 1,139; IC95%: 0,621-1,657; p < 0,001). Las enfermeras que eligieron voluntariamente la profesión presentaron menor fatiga por compasión (49,14 ± 19,39 vs. 61,93 ± 21,42; p = 0,032). Trabajar más de 50 horas semanales aumentó la empatía (84,06 ± 11,58 vs 78,82 ± 6,46; p=0,046) pero no se relacionó con la fatiga por compasión.

**Conclusión:**

Mayores puntuaciones de empatía se asociaron con mayores puntuaciones de fatiga por compasión en personal de enfermería de unidades de cuidados intensivos neonatales/pediátricos . La elección voluntaria de la profesión se relacionó con menor fatiga por compasión, mientras que las jornadas laborales prolongadas se asociaron con mayor empatía.

## INTRODUCTION

Empathy and compassion are central components of nursing care, because they support an understanding of patients’ experiences and the development of responsive therapeutic relationships^1^^,^^2^. Empathy is defined as an individual’s ability to understand and share the experiences, feelings, and perspectives of others^3^. In nursing, empathy is known to strengthen therapeutic communication, improve the nurse - patient relationship, and increase patient satisfaction. Compassion, by contrast, is characterized by a more affective response, including emotional resonance with a patient’s suffering and a motivation to alleviate it^1^. Compassion fatigue is characterized by emotional exhaustion and a reduced capacity for compassion resulting from prolonged exposure to traumatic or stressful care situations. It can lead to psychological problems such as anxiety, depression, and sleep disorders, and negatively impact job satisfaction, professional commitment and the quality of care^4^.

Within healthcare, certain clinical environments place particularly intense emotional demands on nurses. Critical care, oncology, palliative care, and emergency settings expose nurses to ongoing patient suffering, frequent ethical decision-making, recurrent end-of-life encounters, and complex communication with patients’ families^5^^,^^6^. Among these, neonatal and pediatric intensive care units (NICUs and PICUs) represent a distinct clinical context. Nurses working in these units provide care for vulnerable infants and children in life-threatening situations who are physiologically fragile and may experience rapid clinical deterioration, and often require continuous monitoring and highly specialized care. Mortality rates in these units are higher than in many adult care settings, leading to repeated exposure to loss. Furthermore, while also providing families with intense emotional support, nurses must simultaneously care for the children and their families, with an additional emotional layer compared to that in only adult services. This process requires continuous empathic interaction and a high level of emotional involvement. However, when experienced intensely and over an extended period, empathy may contribute to compassion fatigue resulting from constant exposure to the suffering of others^4^^,^^7^^,^^8^.

Despite growing recognition of compassion fatigue as a significant issue in nursing, comparatively few studies have jointly examined empathy and compassion fatigue specifically among NICUs and PICUs nurses. Previous research have assessed empathy and compassion fatigue in adult intensive care, oncology, and emergency settings ^3^^,^^5^^,^^9^^-^^11^. However, pediatric and neonatal intensive care populations are underrepresented in the literature. Moreover, the relationship between empathy and compassion fatigue remains complex. Some studies have reported a positive association^9^^,^^12^^,^^13^, whereas others suggest that the relationship may depend on factors such as empathy-related guilt, self-compassion, emotional regulation, and organizational support.

Understanding the relationship between empathy and compassion fatigue is particularly important for nurses in these highly demanding intensive care environments, NICUs and PICUs. Maintaining emotional balance and professional well-being is crucial. Therefore, a clearer understanding of the relationship between empathy and compassion fatigue among NICUs and PICUs nurses may help identify nurses who are vulnerable to emotional distress and contribute to the development of appropriate preventive individual strategies -such as emotional regulation and mindfulness practices- and organizational support systems. 

This study aimed to investigate the relationship between levels of empathy and compassion fatigue among nurses working in NICUs and PICUs and their relationship with the sociodemographic and professional characteristics of these nurses. 

## METHODS 

### Study Design and Participants

A descriptive-correlational study was conducted between 1 June and 14 December 2023 in the NICUs and PICUs of three public tertiary hospitals in Tekirdağ, Turkey, including one training and research hospital and two state hospitals. All three centers provide advanced neonatal and pediatric intensive care services. Nurses were eligible to participate if they were employed in the participating hospitals, had at least one year of experience in their current intensive care unit, were actively working in the relevant unit during the data collection period, and voluntarily agreed to participate by providing informed consent. Nurses who were on sick leave, annual leave, or administrative leave were excluded. Nurses who declined participation or withdrew their consent were also excluded. 

No sampling was used, as the aim was to include the entire population of 72 nurses working in the hospitals where the research was conducted.

Ethical approval for the study was obtained from the Social and Human Sciences Research Ethics Committee of Istanbul University - Cerrahpaşa Rectorate (Decision No. 2022/489, dated December 19, 2022). Institutional approval was obtained from each participating hospital. Permission to use the study instruments (scales) was also obtained from the respective copyright holders. Prior to data collection, all eligible nurses received verbal and written information regarding the study objectives and procedures, and written and verbal informed consent was obtained.

### Data collection

The researchers created a form containing seven questions on demographics and ten on professional characteristics. The previously developed and validated Jefferson Scale of Empathy and Compassion Fatigue - Short Scale were also used. All questionnaires were administered face to face.

- Sociodemographic variables included age (years), sex (female or male), highest educational qualification (associate, bachelor´s or master´s degree), marital status (married or single), and having children (yes/no).
- Self-reported personal characteristics were assessed using two researcher-developed questions describing either a predominantly positive (“I am always grateful for what I have and can find something to be happy about”) or negative (“I think negative things always happen to me; I consider myself unlucky”) outlook. Personal characteristic categories (yes, no). These categories (negative, positive, extraverted, and indifferent) were used researchers for descriptive purposes only and do not represent validated personality dimensions or personality types.
- Occupational characteristics included total professional experience (months), current intensive care unit (NICU, PICU), duration of employment in the current unit (months), weather nursing had been chosen voluntarily (yes, no), work schedule (day shift only or rotating day and night shifts), weekly working hours (30-39, 40-49, ≥ 50), satisfaction with the work environment (yes, no), considering transferring to another clinical unit (yes, no), and perceived workload (never, sometimes, often, or always).
- Empathy was assessed using the 20-item Health Professional version of the Jefferson Scale of Empathy. Each item is scored on a seven-point Likert scale ranging from 1 (strongly disagree) to 7 (strongly agree). Ten positively worded items are scored directly, whereas ten negatively worded items are reverse-scored. Total scores range from 20 to 140, with higher scores indicating greater empathy.
- Compassion fatigue was assessed using the 13-item Compassion Fatigue-Short Scale^14^, which was validated for use in Turkish by Yıldırım and Cavcav^15^. Each item is rated on a 10-point Likert scale ranging from 1 (never or rarely) to 10 (very often). The scale comprises two sub-scales: occupational burnout and secondary traumatic stress. There is no cut-off point for the scale, which has a total score range of 13-130; higher scores indicate greater levels of compassion fatigue.

## Data analysis

Categorical variables are presented using frequencies and percentages. Continuous variables are presented using means, standard deviations (SD), and minimum and maximum values. 

The internal consistency of the Jefferson Scale of Empathy and the Compassion Fatigue-Short Scale was evaluated using Cronbach’s alpha coefficients.

The distributions of the empathy and compassion fatigue total scores were assessed using skewness and kurtosis coefficients, were values between -1.5 and +1.5 were considered to indicate no substantial departure from normality)^16^. In such cases, scores were described by mean and SD and compared using parametric statistical methods. Subgroup-specific Shapiro-Wilk and Levene test results were not available in the finalized statistical output; therefore, comparisons involving small subgroups were interpreted cautiously.

Scale scores were compared between two independent groups using independent-samples *t*-tests and among three or more groups using ANOVA. In the event of significant results, *post-hoc* pairwise comparisons were performed using Tukey’s honestly significant difference and Fisher’s least significant difference tests. Given the relatively small sample sizes in some subgroups, these *post-hoc* findings were interpreted cautiously.

The relationship between empathy and compassion fatigue scores was examined not only using Pearson’s r correlation coefficient but also via univariate linear regression to determine the unstandardized regression coefficient B, its 95% confidence interval (95% CI), and the coefficient of determination (R^2^). The Durbin-Watson statistic was reported as a residual diagnostic and was interpreted cautiously because the data were cross-sectional.

Data were analyzed using IBM SPSS Statistics for Windows, Version 22.0. All statistical tests were two-sided, and statistical significance was set at *p* < 0.05.

## RESULTS

Sixty-five nurses across three hospitals volunteered to participate in the study (representing 90.28% of the target population).

The mean age of the participating nurses was 31.88 years (range: 23-47). The vast majority were female (98.5%), and most held a bachelor’s degree (75.4%). In terms of personal demographics, 55.4% were married and 44.6% had children. Regarding clinical units, 63.1% worked in a NICU. Most nurses reported having voluntarily chosen both the nursing profession and their current unit (76.9%). The majority worked rotating day and night shifts (89.2%), and 50.8% worked 40-49 hours per week. Overall, 63.1% were satisfied with their work environment, whereas 32.3% reported considering a transfer to another clinic (Table 1).

**Table 1:** Demographic and occupational characteristics of the participating nurses

Characteristics			
Demographic	n (%)	Occupational	n (%)
Gender (women)	64 (98.5)	Length of experience (months), *mean±SD*	111.65 ± 85.58
Age (years), *mean±SD*	31.88 ± 7.19	Intensive care unit	
Educational level		Neonatal	41 (63.1)
Associate degree	8 (12.3)	Pediatric	24 (36.9)
Bachelor’s degree	49 (55.4)	Length in current ICU (months), *mean±SD*	53.95 ± 42.12
Master’s Degree	8 (12,3)		
Marital status		Willingly Choosing	
Married	36 (55.4)	Nursing Profession	50 (76.9)
Single	29 (44.6)	Current Unit	50 (76.9)
Having Children	29 (44.6)	Work Schedule	
Self-Description		Day shift only	7 (10.8)
“I am always grateful for what I have and can find something to be happy about”	60 (92.3)	Day and night shifts	58 (89.2)
		Weekly Working Hours	
		30-39 hours	15 (23.1)
		40-49 hours	33 (50.8)
“I think negative things always happen to me; I consider myself unlucky”	5 (7.7)	≥ 50 hours	17 (26.2)
		Satisfaction with work environment	41 (63.1)
		Considering transfer to another clinic	21 (32.3)
Self-reported personal characteristics		Overwhelmed by job responsibilities	
Negative	26 (40.0)	Never	3 (4.6)
Positive	53 (81.5)	Sometimes	13 (20.0)
Extraverted	31 (47.7)	Often	40 (61.5)
Indifferent	7 (10.8)	Always	9 (13.8)

SD: standard deviation; ICU: intensive care unit.

The mean length of professional experience was 111.65 ± 85.58 months, and the mean duration of employment in the current ICU was 53.95 ± 42.12 months. The most frequent self-reported personal characteristic was positive (81.5%), and the least frequent was indifferent (10.8%). Because participants could endorse more than one category, these percentages were not mutually exclusive (Table 1).

Sub-analyses by sex were not attempted due to very low percentage of male participants.

The skewness and kurtosis coefficients for empathy (0.612 and 0.476, respectively) and for compassion fatigue (0.343 and −0.053, respectively) were within the pre-specified range; thus, a normal distribution was assumed.

The mean Jefferson Scale of Empathy score was 79.77 ± 8.69 (range: 64-105), which is close to the theoretical midpoint of the scale (80) rather than being clearly low or high. The mean compassion fatigue score was 52.09 ± 20.44, indicating notable variability in compassion fatigue scores among nurses. Because neither scale provides validated cut-off points for this population, these findings should be interpreted as continuous scale scores.

The Cronbach’s alpha coefficients indicated a good level of internal consistency for both the Jefferson Scale of Empathy (α = 0.842) and the Compassion Fatigue-Short Scale (α = 0.85). 

Empathy scores differed significantly only across weekly working-hour categories. Nurses working 50 hours or more per week had higher mean empathy scores (84.06 ± 11.58) than those working 30 - 39 (77.00 ± 7.96) and 40-49 hours per week (78.82 ± 6.46). However, given the relatively small sample sizes in two of the subgroups, these *post-hoc* findings should be interpreted cautiously (Table 2). 

Compassion fatigue scores were significantly lower among nurses who had voluntarily chosen the nursing profession than among nurses who had not chosen it voluntarily (49.14 ± 19.39 vs. 61.93 ± 21.42; p = 0.032) (Table 2).

**Table 2:** Comparison of empathy and compassion fatigue scores according to descriptive characteristics

Descriptive characteristics	n	Empathy (Mean ± SD)	Compassion Fatigue (Mean ± SD)
Education level			
Associate degree	8	81.375±11.661	59.375±18.244
Bachelor’s degree	49	79.837±8.533	50.510±20.897
Master’s degree	8	77.750±6.840	54.500±20.164
F		0.347	0.704
p		0.708	0.499
Marital status			
Married	36	79.306±9.783	51.972±23.200
Single	29	80.345±7.222	52.241±16.786
T		-0.477	-0.052
p		0.635	0.957
Having children			
Yes	29	79.035±9.206	54.862±23.585
No	36	80.361±8.326	49.861±17.527
T		-0.609	0.980
p		0.545	0.331
Unit of employment			
Neonatal ıntensive care unit	41	79.537±8.028	53.683±18.540
Pediatric ıntensive care unit	24	80.167±9.876	49.375±23.498
T		-0.280	0.818
p		0.780	0.416
Choosing the nursing profession			
Willingly	50	78.980±8.508	49.140±19.387
Unwillingly	15	82.400±9.046	61.933±21.423
T		-1.346	-2.188
p		0.183	0.032
Choosing the current unit			
Willingly	50	79.360±8.297	52.960±19.537
Unwillingly	15	81.133±10.063	49.200±23.698
T		-0.691	0.622
p		0.492	0.536
Weekly working hours			
30-39 hours (group 1)	15	77.000±7.964	47.333±19.397
40-49 hours (group 2)	33	78.818±6.464	52.242±16.287
≥ 50 hours (group 3)	17	84.059±11.578	56.000±27.810
F		3.247	0.712
p		0.046	0.495
Tuckey HSD		g3 > (g1 & g2)	
Satisfaction with work environment			
Yes	41	80.073±8.107	51.366±19.662
No	24	79.250±9.755	53.333±22.074
t		0.366	-0.372
p		0.715	0.711
Considering transfer to another clinic			
Yes	21	79.571±10.308	56.476±26.581
No	44	79.864±7.926	50.000±16.707
t		-0.126	1.199
p		0.900	0.315
		Pearson’s r (p)	
Age (years)	65	-0.170 (0.176)	-0.035 (0.780)
Length of professional experience (months)	65	-0.063 (0.619)	0.115 (0.363)
Length of work in current ICU (months)	65	-0.035 (0.780)	0.202 (0.106)

SD: standard deviation; ICU: intensive care unit; F: ANOVA; t: independent-samples t test; r: Pearson’s correlation coefficient; Tukey HSD: Tukey’s honestly significant difference *post-hoc* test; g: group.

No statistically significant differences in empathy or compassion fatigue scores were found according to any other studied variable.

Empathy showed a moderate, significant positive association with compassion fatigue (r = 0.484, *p* < 0.01). Given the sample size (n = 65), a two-tailed α = 0.05, and an observed r = 0.484, the achieved statistical power (calculated via G*Power 3.1) was 0.987. Empathy explained 22.2% of the variance in compassion fatigue scores according to the overall lineal regression model. Each one-point increase in empathy was associated with an estimated 1.139-point increase in compassion fatigue (B = 1.139, 95% CI: 0.621-1.657; *p* < 0.001). The Durbin-Watson statistic (1.148) may indicate some residual dependence; thus, these regression findings were interpreted cautiously. 

This significant relationship between empathy and compassion fatigue was not observed when evaluating subgroups based on weekly working hours or whether the nursing profession was voluntarily chosen. Although compassion fatigue scores increased numerically across the weekly working-hour categories, the overall difference was not statistically significant. Empathy scores were slightly higher when the profession was chosen involuntarily (Table 2).

## DISCUSSION 

In this study we examined the relationship between empathy levels and compassion fatigue among nurses working in NICUs and PICUs. The two scores moderately correlate, while the only significant relationship with other variables are empathy weekly working-hour categories and compassion fatigue and whether nursing had been voluntarily chosen as a profession. The lack of significant relationships with most other variables may be influenced by the small size in some subgroups.

Nurses have high empathic tendencies^11^^,^^17^ and higher empathy levels have been reported among nurses working in internal medicine and surgical wards compared to those working in emergency departments and ICUs^11^.

Compassion fatigue is present to a moderate to high degree among nurses working in emotionally demanding units such as ICUs^18^^,^^19^, as well as in pediatrics, oncology, palliative care, and emergency services^5^^,^^6^^,^^10^. Similarly, midwives working in delivery rooms have equated compassion with empathy and experienced compassion fatigue^20^. A significant proportion of nurses working in these units experience or are at risk of compassion fatigue^21^^,^^22^.

No significant differences are seen in empathy and compassion fatigue scores according to age, which is in line with previous findings^7^^,^^23^. However, studies involving pediatric nurses reported that compassion fatigue is more prevalent among nurses younger than 40 years and among those with 6-10 years of experience^6^^,^^24^^,^^25^. 

There is no relationship with marital status or number of children, as in the study by Tanrıkulu and Ceylan’s^23^. Similarly, no significant relationships are observed according to whether respondents have children, the unit in which they worke, satisfaction with the work environment, or intention to transfer to another clinic.

Our study found no differences in compassion fatigue scores according to education level, consistent with the study by Branch and Klinkenberg^7^. However, other studies have reported that ward nurses with higher education levels had higher empathy scores^17^ and that compassion fatigue decreased as education level increased^8^^,^^26^^,^^27^. 

This study found no direct relationship between nurses’ professional experience and empathy or compassion fatigue in accordance with previous findings^7^. While some studies report that empathic tendencies increase with professional experience^28^, others have found that compassion fatigue is higher among healthcare professionals with longer-term experience^26^. These findings suggest that the relationship of age and professional experience with empathy and compassion fatigue may vary depending on the context.

This study found that nurses who had chosen their profession willingly had lower compassion fatigue scores as reported by Tanrıkulu and Ceylan^19^ and Şahin *et al*.^23^. Furthermore, research has shown that job satisfaction and liking one’s profession significantly affect compassion fatigue^29^. This may be because nurses who consciously choose their profession are better prepared for professional responsibilities and potential challenges.

In this sample, nurses working 50 hours or more per week have higher empathy scores than those working fewer hours. This finding differs from previous studies reporting lower empathy with increasing weekly working hours^11^^,^^30^. This discrepancy may reflect differences in clinical context, staffing patterns, workload distribution, or characteristics of nurses who undertake longer working hours, aspects that were not analyzed here. Given the cross-sectional design and the limited subgroup size, this finding should be regarded as exploratory. Although other studies have reported associations between compassion fatigue and longer working hours^8^^,^^19^, in the present study compassion fatigue scores increase numerically across working-hour categories without reaching statistical significance. This finding may have been influenced by the small size of some categories. 

Higher empathy scores were associated with higher compassion fatigue scores in this study. Similarly, studies conducted with nurses and nursing students have reported associations between higher empathic tendencies and greater compassion fatigue^1^^,^^9^^,^^31^, while a study of pediatric nurses found that empathy was more strongly associated with compassion fatigue than with secondary traumatic stress and burnout^12^. Previous studies have identified empathy as an influential factor in the development of compassion fatigue, however, the direction and strength of this relationship remain unclear^32^^,^^33^. 

This study has several limitations that may affect the interpretation of the findings. The study was conducted among PICUs and NICUs nurses from three hospitals in a single province in Turkey, which may limit the external validity of the results. Despite approximately 90% of the nursing personnel participating in the study, the sample size was limited. This mainly affects subgroup analyses, some of which were based on relatively small and unequal group sizes, particularly for weekly working hours and critical care unit distribution. Because of the cross-sectional design, changes in empathy and compassion fatigue over time could not be evaluated and causal inferences cannot be made. Moreover, all data were collected using self-report questionnaires. Participants’ personal characteristics were assessed using researcher-developed binary categories rather than a validated multidimensional personality instrument. The Durbin-Watson statistic was 1.148. However, because the data were cross-sectional and had no inherent temporal ordering, the diagnostic value of this statistic was limited. Accordingly, the regression findings should be interpreted cautiously, as the associations observed within this sample. Taken together, these findings suggest that empathy and compassion fatigue may be related; however, the cross-sectional design, limited sample size, and uneven subgroup distribution do not allow conclusions regarding causality or the direction of this relationship.

In conclusion, nurses in pediatric and neonatal critical care units demonstrated moderate to- favorable empathy levels. Working 50 hours or more per week associates with higher empathy scores, voluntarily choosing nursing associates with lower compassion fatigue scores, and higher empathy scores associates with greater compassion fatigue. Future research should examine supportive strategies to prevent compassion fatigue, particularly among nurses with higher empathy scores.

Given the positive association observed between empathy and compassion fatigue among nurses working in NICUs and PICUs, routine assessment of compassion fatigue and institutional support for nurses with higher empathy scores may be considered in clinical practice. Strategies such as reviewing nurses’ working-hour arrangements, enabling them to work in preferred clinical settings where feasible and improving access to psychosocial support resources may help reduce the risk of compassion fatigue.

## Data Availability

The data that support the findings of this study are available on request from the corresponding author. The data are not publicly available due to privacy or ethical restrictions.
